# Pre-stimulus Brain Activity Is Associated With State-Anxiety Changes During Single-Session Transcranial Direct Current Stimulation

**DOI:** 10.3389/fnhum.2019.00266

**Published:** 2019-08-08

**Authors:** Keiichiro Nishida, Yosuke Koshikawa, Yosuke Morishima, Masafumi Yoshimura, Koji Katsura, Satsuki Ueda, Shunichiro Ikeda, Ryouhei Ishii, Roberto Pascual-Marqui, Toshihiko Kinoshita

**Affiliations:** ^1^Department of Neuropsychiatry, Kansai Medical University, Osaka, Japan; ^2^Division of Systems Neuroscience of Psychopathology, Translational Research Center, University Hospital of Psychiatry, University of Bern, Bern, Switzerland; ^3^Osaka Prefecture University Graduate School of Comprehensive Rehabilitation, Osaka University, Osaka, Japan; ^4^The KEY Institute for Brain-Mind Research, University of Zurich, Zurich, Switzerland

**Keywords:** transcranial direct current stimulation, left dorsolateral prefrontal cortex, dorsomedial prefrontal cortex, anterior cingulate, anxiety, depression

## Abstract

Transcranial direct current stimulation is a promising neuromodulation method for treating depression. However, compared with pharmacological treatment, previous studies have reported that a relatively limited proportion of patients respond to tDCS treatment. In addition, the neurophysiological mechanisms underlying tDCS treatment remain unclear, making it difficult to identify response predictors for tDCS treatment based on neurophysiological function. Because treatment effects are achieved by repetitive application of tDCS, studying the immediate effects of tDCS in depressive patients could extend understanding of its treatment mechanisms. However, immediate changes in a single session of tDCS are not well documented. Thus, in the current study, we focused on the immediate impact of tDCS and its association with pre-stimulus brain activity. To address this question, we applied anodal tDCS to the left dorsolateral prefrontal cortex (DLPFC) or dorsomedial prefrontal cortex (DMPFC) in 14 patients with major depressive disorder (MDD) and 19 healthy controls (HCs), at an intensity of 1.0 mA for 20 min in a single session. To evaluate anxiety, the state trait anxiety inventory was completed before and after tDCS. We recorded resting electroencephalography before tDCS, and calculated electrical neuronal activity in the theta and alpha frequency bands using standardized low-resolution electromagnetic tomography. We found that, during application of left DLPFC tDCS to patients with MDD, the anxiety reduction effect of tDCS was related to higher baseline theta-band activity in the rostral anterior cingulate cortex (rACC) and no medication with benzodiazepine used as hypnotic. For DMPFC stimulation in MDD, the anxiety reduction effect was associated with lower baseline alpha-band activity in the left inferior parietal lobule. In contrast, in HCs, the anxiety reduction effect was associated with higher baseline alpha activity in the precuneus during DMPFC stimulation. The current results suggest that the association between pre-tDCS brain activity and the anxiety reduction effect of tDCS depends on psychopathology (depressed or non-depressed) as well as the site of stimulation (DMPFC or left DLPFC) and insomnia. Furthermore, the results suggest that tDCS response might be associated with baseline resting state electrophysiological neural activity.

## Introduction

Transcranial direct current stimulation is a widely used neuromodulation technique for basic neurocognitive research in healthy subjects as well as clinical applications in major depression and other psychiatric disorders (Fregni et al., 2015; Martin et al., 2018) In clinical practice, the development of new treatment approaches without medication is important for patients, who show low tolerance to pharmacotherapy because of substantial side effects (Brunoni et al., 2012). tDCS provides a potentially useful approach because the tDCS stimulator is a mechanically simple device, with a lower cost than other non-invasive brain stimulation devices.

In recent decades, major depressive disorder (MDD) has become one of the most serious lifetime diseases in many countries (Murray and Lopez, 1996). Although treatments for MDD have improved, current treatment options have limitations (Kupfer et al., 2012).

In treatment methods involving non-invasive brain stimulation for MDD, the left dorsolateral prefrontal cortex (DLPFC) has been found to play a major role in executive functioning, and is widely recognized as a suitable target for anodal tDCS to recover executive control and emotion regulation.

A recent meta-analysis supports the application of tDCS to the DLPFC in MDD (Mutz et al., 2018). Furthermore, a recent large-scale study reported no inferiority of tDCS treatment compared with escitalopram (Brunoni et al., 2017). However, the specific treatment effects of tDCS remain controversial (Tremblay et al., 2014; Mondino et al., 2015; Brunoni et al., 2017; Martin et al., 2018). A recent study by Brunoni and colleagues reported that response rates to tDCS were significantly higher than placebo, but the remission rate was not significantly different between tDCS and placebo groups (Brunoni et al., 2017). Furthermore, the treatment mechanisms of tDCS remain unclear.

Recently, several studies proposed additional targets for treatment of MDD, suggesting non-invasive brain stimulation of the dorsomedial prefrontal cortex (DMPFC) as one potential approach (Downar and Daskalakis, 2013). This proposal is based on the finding that the DMPFC, including the anterior cingulate cortex, is involved in regulation of emotions (Bush et al., 2000), and is anatomically connected with the amygdala and nucleus accumbens, which have both been implicated in MDD.

More recent studies have confirmed the feasibility of rTMS on the DMPFC for MDD (Downar et al., 2014; Kreuzer et al., 2015; Schulze et al., 2018). However, we know little evidence of the mechanism of DMPFC stimulation even beyond the context of major depression (Bakker et al., 2015; Colzato et al., 2015; Kreuzer et al., 2015).

There are several limitations of the current evidence supporting further implementation of tDCS into clinical practice. First, better understanding of the neurophysiological mechanisms underlying the effects of tDCS is needed. Second, biomarkers are needed for predicting tDCS treatment responders. One possible approach for addressing these current limitations is to examine the neurophysiological signatures of patients. Specifically, the pre-stimulus state of the brain may explain the variability in responses to tDCS. Recent studies have reported that pre-treatment electroencephalography (EEG) predicts changes in cognition after 15 sessions of tDCS in the left DLPFC in depressive patients, and that frontal electrodes exhibit predictive power for changes in cognition (Al-Kaysi et al., 2016, 2017).

Although predicting treatment effects with pre-treatment neurophysiological activity would have direct implications for clinical practice, the neurophysiological mechanisms underlying treatment effects may remain obscured because treatment effects are achieved by repeated application of single-session tDCS, and the accumulation of immediate neural responses to single-session tDCS may modify the stable state of brain activity and eventually improve depressive symptoms. Therefore, we assumed that examining the neural mechanisms of a single-session of tDCS intervention might provide a first step for disentangling the complex treatment mechanisms of tDCS for MDD. Among symptomatic problems of MDD, single session of tDCS is hard to change sustained symptoms such as depressive mood, anhedonia, agitation or loss of motivation, while anxiety is relatively volatile across time. In the current study, we therefore focus on state anxiety to look at the effect of single-session tDCS.

In the current study, we set two main aims. First, we investigated the immediate effects of prefrontal tDCS on brain activity and state anxiety. Second, we compared the effects between stimulation of the left DLPFC, the canonical target of tDCS for MDD, and the DMFPC, the potential target predicted by neuroanatomical architecture. To this end, we applied anodal tDCS to the left DLPFC or DMPFC in MDD patients and healthy controls (HCs), with 20 min of stimulation in a single session. We measured state anxiety before and after tDCS and neural activity with EEG before tDCS. Finally, we examined association between neural activity and state anxiety to investigate neural predictors of the change in anxiety induced by tDCS.

## MATERIALS AND METHODS

### Participants

We recorded a total of 20 patients with MDD, assessed by the DSM-IV and evaluated with the Hamilton Depression Rating Scale (HAM-D), and a total 24 HCs subjects recruited for this study. After eliminating data corresponding to subjects that were left-handed, or unavailable EEG, or psychological evaluation, 14 patients with MDD and 19 HCs were finally included in this study. All participants were right-handed, and were graduates of high school or higher education. All participants were diagnosed by experienced psychiatrists using a structured interview and physical examination. We excluded patients with history of dementia, schizophrenia, substance dependence, epilepsy or head trauma. Participants do not have anxiety disorder comorbidities, such as generalized anxiety disorder, panic disorder, and phobia. Thirteen patients have received antidepressant. 10 patients were medicated by benzodiazepine as sleeping medication and 2 patients medicated by a mood stabilizer. Chi-squared test with Yates’s correction between gender did not show significant difference (*x*^2^(1) = 3.076, *p* (0.08). All HCs had no history of psychiatry disorders. This study was carried out in accordance with the recommendations of “Safety of transcranial direct current stimulation, tDCS by Japanese Journal of Clinical Neurophysiology 2011.”

The study protocol was approved by the Institutional Ethics Review Committee of Kansai Medical University (UMIN000015046). We obtained written informed consent from all participants in accordance with the Declaration of Helsinki. Participants were recruited from September 2014 to April 2017. Details of participants are shown in Table 1.

**TABLE 1:** Demographic data.

**Group**	**MDD**		**HC**	
**Session**	**Left DLPFC**	**DMPFC**	**Left DLPFC**	**DMPFC**
Sample size	14		19	
Sex: Male/female	12/2		12/7	
**Drug treatment**				
No	1		0	
One antidepressant	11		0	
Two antidepressants	2		0	
Benzodiazepine	10		0	
Mood stabilizer	2		0	
Age-years: mean ± SD	44.93 ± 14.68		48.94 ± 15.80	
Education period	15.36 ± 1.55		15.63 ± 1.34	
Number of previous episodes: mean ± SD	2.36 ± 0.93		0.00 ± 0.00	
HAM-D17 score on the day of the session: mean ± SD	14.07 ± 5.40	13.79 ± 4.82	0.21 ± 0.42	0.21 ± 0.54

### tDCS

**FIGURE 1:**
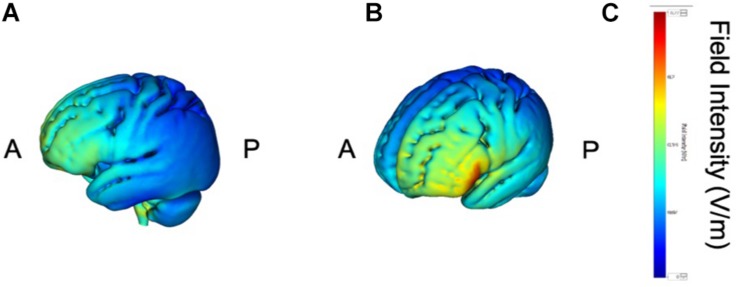
Modeling of electric field distribution for the montage of left DLPFC stimulation. **(A)** Sagittal view, **(B)** side view, **(C)** above view, A: anterior, P: posterior.

**FIGURE 2:**
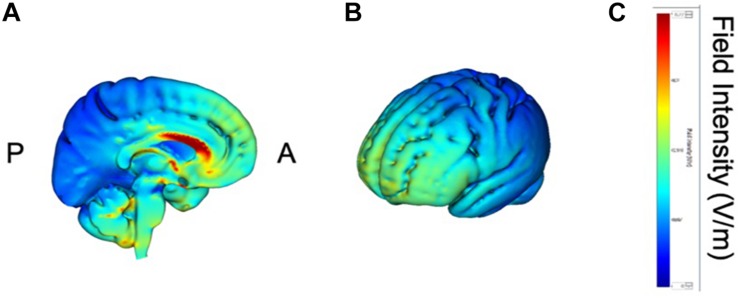
Modeling of electric field distribution for the montage of DMPFC stimulation. **(A)** Sagittal view, **(B)** side view, **(C)** above view, A: anterior, P: posterior.

tDCS was administered with a battery-driven stimulator (DC Stimulator Plus, Neuroconn, Ilmenau, Germany). The electrical current was applied at 1 mA via electrically conductive rubber electrodes (20 cm^2^, circular in shape) attached with an adhesive conductive EEG paste. Anodal stimulation was administered over the left-DLPFC (F5, 10–10 EEG international electrode placement, Figure 1) or the DMPFC (AFz, 10-10 EEG international electrode placement, Figure 2) with the cathodal electrode placed on the left shoulder. Direct current was administered for 20 min during the resting state. We also simulated the current flow of our montage with a simulation software using a finite element model (HD-explore, Soterix Medical, New York, United States) (Figures 1, 2).

### Procedure

**FIGURE 3:**
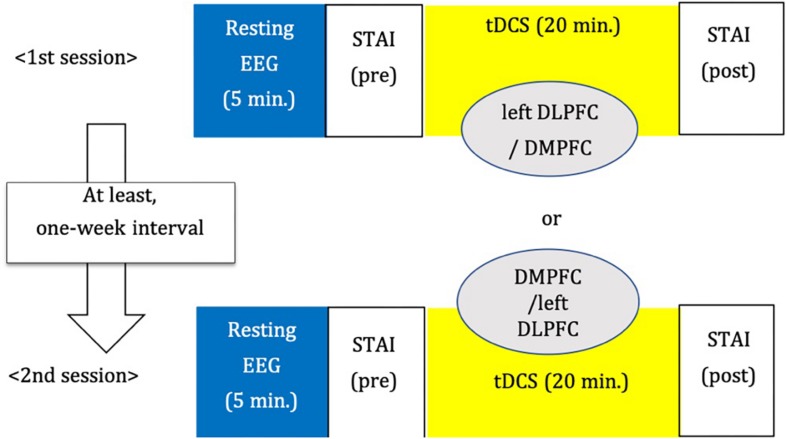
Study design.

We adopted a between-subjects cross-over design (Figure 3). The order of stimulation was counterbalanced. Each subject was randomly assigned to receive left DLPFC or DMPFC tDCS in the first session. The participant received tDCS on the other site in the second session. There was an interval of at least 1 week between tDCS sessions.

### Psychological Test

We measured the STAI (state-trait anxiety inventory), which consists of two subscales, STAI-SA for state anxiety to assess anxiety before and after tDCS and STAI-TA for trait anxiety before tDCS.

### EEG Recording

Resting and eyes-closed EEG was recorded with an EEG-1200 Nihon Kohden (Tokyo, Japan) system. A 64 ch Ag/AgCl sintered Waveguard Original EEG cap from ANT Neuro (Netherland) was used for the recordings. It was necessary to use a subset of the electrodes comprising of 19 EEG electrodes corresponding to the international 10–20 system for analyses, because the tDCS electrodes placed under the EEG cap interfered with substantial number of EEG electrodes in the frontal area. We recorded EEG before and after tDCS. However, only EEG recordings before tDCS were used in the analyses described below.

### EEG Analysis

Signals of cortical electric neuronal activity were computed from the baseline, pre-stimulation EEG recordings using standardized low resolution electromagnetic tomography (sLORETA) (Pascual-Marqui, 2002). In its current implementation (free academic software package available at https://www.uzh.ch/keyinst/loreta), this method produces signals of appropriately standardized current density from 6239 cortical gray matter voxels, sampled on a 5 mm resolution grid, using the MNI152 anatomical template (Mazziotta et al., 2001; Fuchs et al., 2002). sLORETA has received both theoretical (Greenblatt et al., 2005; Sekihara et al., 2005; Pascual-Marqui, 2007) and experimental validation (Pascual-Marqui et al., 2009).

The sLORETA signals were then further processed to produce values of cortical spectral power in two classical EEG frequency bands: theta (4–8 Hz) and alpha (8–12 Hz). We chose these two frequency bands because they have been repeatedly reported to be associated with MDD and response to treatment (Klimesch, 1999; Moore et al., 2000; Nishida et al., 2015; Kitaura et al., 2017).

### Regions of Interest

**FIGURE 4:**
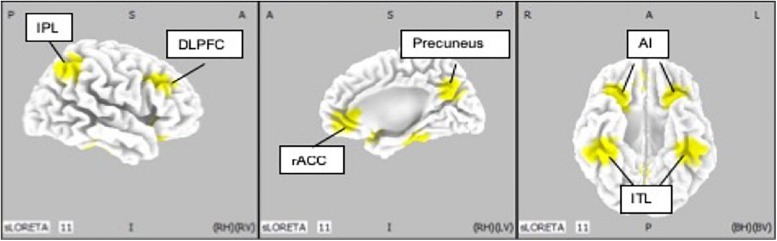
Location of regions of interest (IP: inferior parietal lobe; DLPFC: dorsolateral prefrontal cortex; rACC: rostral anterior cingulate cortex; AI: anterior insula; ITL: inferior temporal lobe).

Ten regions of interest (ROIs) were chosen based on previous studies investigating neurophysiological mechanisms in patients with MDD (McGrath et al., 2013; Kaiser et al., 2015; Pizzagalli et al., 2018) (Figure 4). Pizzagalli et al., investigated the importance of current density in rACC for improvement of depression symptoms with EEG-LORETA. The meta-analysis by Kaiser et al., showed significant difference in resting state functional connectivity in patients with depression and HCs. In addition, McGrath et al. have shown that anterior insula and inferior temporal lobe were candidates of biomarkers of treatment by cognitive behavior therapy by using positron emission topography.

We defined rACC from average coordinates (i.e., centroid) of the atlas which Pizaggali et al. used in their paper. Atlas of DLPFC and Inferior temporal lobe in our study was obtained from Kaiser’s literature, and the coordinates used for the anterior insula and inferior temporal lobe originate from the work of McGrath (McGrath et al., 2013; Table 2).

**TABLE 2:** Regions of interest (ROIs) and their coordinates in the MNI space.

	***x***	***y***	***z***
rACC	0	45	0
Left DLPFC	−40	26	34
Right DLPFC	40	26	34
Left AI	−30	24	−13.5
Right AI	30	24	−13.5
Left ITL	−42	−33	−25.5
Right ITL	42	−33	−25.5
Left IPL	−45	−52	48
Right IPL	45	−52	48
Precuneus	0	−66	34

*rACC, rostral anterior cingulate cortex; DLPFC, dorsolateral prefrontal cortex; AI, anterior insula; ITL, inferior temporal lobe; IPL, inferior parietal lobe.*

### Statistical Analysis

For each stimulation session, the change in anxiety scores was defined as the STAI-SA score at post tDCS minus the STAI-SA score at pre tDCS (baseline). Thus, a negative value of the change indicates a reduction of state anxiety. In the current study, we aimed to investigate the association between these dependent variables and cortical activities in 10 ROIs. We also included the “with” or “without” administration of benzodiazepines as an independent variable, and baseline STAI-SA scores for considering the effect of the diversity of participants.

Firstly, we applied a least absolute shrinkage and selection operator (LASSO) for selecting appropriated variables and regularization. The set of independent variables consisted of the cortical spectral power for the theta and alpha bands, at 10 ROIs calculated from baseline, pre-stimulation EEG-sLORETA for each DLPFC or DMPFC session, plus the medication about with/without benzodiazepines and STAI-SA scores at pre-tDCS in patients and controls separately. Cross-Validation leave-one-out was performed to determine the optimal tuning penalty parameter (*λ*) for each session. Finally, variable selection was performed by using the estimated λ value. We performed LASSO with R (3.6.0), RStudio (1.2.1335), and glmnet package (2.0–18).

Next, forced entry multiple regression analyses were conducted for changes in STAI-SA scores as dependent variables, with the set of cortical activity in each theta and alpha band at selected independent variable, for both left DLPFC and DMPFC stimulation session, in the MDD group and in the control group. SPSS version 26 was used for this multiple regression analysis.

### Adverse Events

Six of 23 participants reported headaches, tingling sensation, itching, or experiencing the taste of iron. Because all reported events were mild, all participants continued the experiments and recovered from the adverse effects immediately after the sessions.

## Results

### Change in STAI Scores

We first examined overall changes in state anxiety. Table 3 shows the baseline and the Post–Pre tDCS changes in STAI-SA scores (Table 3).

**TABLE 3:** STAI-SA pre tDCS and STAI-SA post tDCS.

**Group**	**MDD**		**HC**	
**Session**	**Left DLPFC**	**DMPFC**	**Left DLPFC**	**DMPFC**
STAI-SA_pre	47.29 (±9.10)	44.71 (±8.11)	36.32 (±7.02)	35.84 (±6.80)
STAI-SA_post	44.64 (±9.96)	43.14 (±9.81)	38.42 (±6.40)	37.16 (±6.52)
Post–Pre change of STAI-SA	−2.64 (±5.23)	−1.57 (±6.01)	2.11 (±5.86)	1.32 (±4.35)

*DLPFC, dorsolateral prefrontal cortex stimulation; DMPFC: dorsomedial prefrontal cortex stimulation, MDD: major depressive disorder, HC: healthy controls, STAI-SA: State-Trait Anxiety Inventory - state anxiety.*

Analysis of variance revealed that the baseline STAI-SA score was significantly higher in the MDD group than that in the HC group (*F*[3,62] = 8.943, *p* < 0.001). However, analysis of covariance revealed no significant difference in changes of STAI-SA score between the two groups (*F*[1,63] = 1.562, *p* = 0.215), and within each group (MDD: *F*[1,25] = 0.180, *p* = 0.675; HC: *F*[1,35] = 0.397, *p* = 0.533). The paired *t*-test did not show the significantly between the score of pre-tDCS and the one of post-tDCS in both MDD (left DLPFC session: *t* = 1.67, *df* = 14, *p* = 0.12, DMPFC session: *t* = 0.83, *df* = 14, *p* = 0.42) and HC (left DLPFC session: *t* = −1.57, *df* = 18, *p* = 0.14, DMPFC session: *t* = −1.32, *df* = 18, *p* = 0.20) groups.

In order to examine the influence of baseline STAI-SA score on tDCS-induced changes of STAI-SA, we performed single regression analysis where the independent variable is the baseline STAI-SA score for each group, and the dependent variable is as STAI-SA change (Table 4). We did not find significant association between pre-tDCS STAI-SA score and tDCS-induced changes of STAI-SA score in the MDD group (*p* = 0.61), while it was significant in the HC group (β = −0.339, *p* = 0.004). We also examined whether pre tDCS STAI-SA score was different between the subjects treated with benzodiazepine and those without benzodiazepine. Mann–Whitney *U*-tests did not show the significant difference between the two groups (*p* = 0.72).

**TABLE 4:** Single regression analysis about STAI-SA.

**Session**	**Dependent variable**	**Independent variable**	**β**	**SE β**	***t*-value**	***p*-value**	**Standard β**	***R*2**
MDD	Change of STAI-SA	STAI-SA pre	−0.064	0.127	−0.507	0.616	−0.099	−0.028
HC	Change of STAI-SA	STAI-SA pre	−0.339	0.111	−3.047	0.004	−0.453^∗∗^	0.183

*MDD, major depressive disorder; HC, healthy controls; STAI-SA, State-Trait Anxiety Inventory-state anxiety; β, the regression coefficient; SE β, standard error of the regression coefficient; *R*^2^, the squared multiple correlation coefficient.*

### Brain Activity in ROIs

We further examined pre-tDCS brain activity estimated by sLORETA. Table 5 shows the values of brain activity calculated by LORETA. Paired *t*-tests revealed no significant difference in log-transformed current density power between the DLPFC and DMPFC sessions, in each of the MDD and HC groups (MDD: *p* = 0.299; HC: *p* = 0.255).

**TABLE 5:** Log-transformed current density power at 10 ROIs in alpha and theta bands.

**Group**	**MDD**				**HC**			
**Session**	**Left DLPFC**		**DMPFC**		**Left DLPFC**		**DMPFC**	
**Current density**	**Mean**	***SD***	**Mean**	***SD***	**Mean**	***SD***	**Mean**	***SD***
Theta_rACC	0.186	0.518	0.133	0.570	0.521	0.519	0.400	0.454
Theta_leftDLPFC	−0.380	0.459	−0.386	0.411	−0.079	0.526	−0.153	0.417
Theta_rightDLPFC	−0.342	0.512	−0.258	0.480	−0.214	0.492	−0.162	0.462
Theta_leftInsula	0.072	0.528	0.070	0.437	0.478	0.491	0.346	0.361
Theta_rightInsula	0.138	0.585	0.215	0.531	0.276	0.516	0.279	0.447
Theta_lrftITP	−0.350	0.413	−0.246	0.247	−0.003	0.437	−0.084	0.366
Theta_rightITP	−0.328	0.467	−0.199	0.376	−0.115	0.438	−0.264	0.466
Theta_leftIPL	−0.531	0.481	−0.667	0.378	−0.216	0.426	−0.119	0.618
Theta_rightIPL	−0.543	0.486	−0.637	0.433	−0.253	0.481	−0.254	0.606
Theta_precuneus	−0.412	0.669	−0.613	0.521	−0.175	0.467	−0.013	0.582
Alpha_rACC	−0.172	0.399	−0.190	0.331	−0.017	0.406	−0.111	0.358
Alpha_leftDLPFC	−0.720	0.468	−0.661	0.242	−0.484	0.331	−0.508	0.310
Alpha_rightDLPFC	−0.525	0.453	−0.432	0.315	−0.636	0.409	−0.681	0.341
Alpha_leftInsula	−0.056	0.545	0.009	0.230	0.157	0.273	0.039	0.292
Alpha_rightInsula	0.116	0.590	0.185	0.376	−0.028	0.311	−0.099	0.257
Alpha_lrftITP	0.233	0.794	0.535	0.494	0.381	0.614	0.299	0.592
Alpha_rightITP	0.404	0.925	0.621	0.633	0.316	0.616	0.064	0.725
Alpha_leftIPL	−0.010	0.793	0.290	0.705	0.140	0.706	0.327	0.763
Alpha_rightIPL	0.173	0.857	0.460	0.706	0.210	0.612	0.179	0.793
Alpha_precuneus	0.254	0.881	0.483	0.758	0.281	0.710	0.357	0.826

*DLPFC, dorsolateral prefrontal cortex; DMPFC, dorsomedial prefrontal cortex, rACC, rostral anterior cingulate cortex; AI, anterior insula; ITL, inferior temporal lobe; IPL, inferior parietal lobe; MDD, major depressive disorder; HC, healthy controls.*

### Multiple Linear Regression Models

To examine whether pre-tDCS brain activity can be associated with anxiolytic effect, we first performed Lasso regression to select predictor variables of each set of theta or alpha activity (Table 6). Here we also included benzodiazepine medication as a predictor variable to control effect of benzodiazepine medication. We then further performed multiple regression analysis if selected variables was associated with tDCS-induced STAI-SA changes. Table 7 shows the results of forced entry multiple linear regression models. Negative values for STAI change corresponded to a reduction of state anxiety after tDCS.

**TABLE 6:** Least absolute shrinkage and selection operator (LASSO) for selecting appropriated variables and regularization.

**Sessions**	**Dependent variable**	**Independent variable**	**λ**	**β**
MDD on left DLPFC	Change of STAI-SA	Theta rACC	0.199	−0.375
		Benzodiazepine		−0.752
MDD on left DLPFC	Change of STAI-SA	Alpha left ITL	0.227	0.285
		Alpha precuneus		0.187
		Benzodiazepine		−0.414
MDD on DMPFC	Change of STAI-SA	Theta left DLPFC	0.262	−0.199
		Theta right insula		−0.036
		Theta precuneus		0.205
MDD on DMPFC	Change of STAI-SA	Alpha rACC	0.156	−0.018
		Alpha left DLPFC		−0.184
		Alpha right ITL		0.114
		Alpha left IPL		0.544
HC on left DLPFC	Change of STAI-SA	Theta n.s.	0.503	n.s.
HC on left DLPFC	Change of STAI-SA	Alpha n.s.	0.503	n.s.
HC on DMPFC	Change of STAI-SA	Theta n.s.	0.244	n.s.
HC on DMPFC	Change of STAI-SA	Alpha rACC	0.220	0.129
		Alpha precuneus		−0.403

*rACC, rostral anterior cingulate cortex; DLPFC, dorsolateral prefrontal cortex; ITL, inferior temporal lobe; IPL, inferior parietal lobe; DLPFC, dorsolateral prefrontal cortex stimulation; DMPFC stimulation, dorsomedial prefrontal cortex; MDD, major depressive disorder; HC, healthy controls; β, the regression coefficient; λ, tuning penalty parameter; n.s, no significance.*

**TABLE 7:** Regression analysis in each theta and alpha bands at on left DLPFC and MDD in patients and HC.

**Session**	**Dependent variable**	**Independent variable**	**β**	**SE β**	***t*-value**	***p*-value**	**Standard β**	***R***
MDD on left DLPFC	Change of STAI-SA	Theta rACC	−5.912	1.809	−3.268	0.007^∗∗^	−0.586	0.583
		benzodiazepine	−6.281	1.998	−3.144	0.009^∗∗^	−0.563	
MDD on left DLPFC	Change of STAI-SA	Alpha left ITL	2.571	1.915	1.343	0.209	0.390	0.598
		Alpha Precuneus	1.728	1.725	1.002	0.340	0.291	
		benzodiazepine	−4.181	2.039	−2.051	0.067	−0.375	
MDD on DMPFC	Change of STAI-SA	Theta left DLPFC	−6.600	6.328	−1.043	0.321	−0.451	0.327
		Theta right Insula	−0.428	5.022	−0.085	0.934	−0.038	
		Theta Precuneus	5.299	2.768	1.914	0.085	0.460	
MDD on DMPFC	Change of STAI-SA	Alpha rACC	−2.237	3.497	−0.640	0.538	−0.123	0.679
		Alpha left DLPFC	−6.678	4.630	−1.442	0.183	−0.268	
		Alpha right ITL	2.259	1.735	1.302	0.225	0.238	
		Alpha left IPL	5.210	1.587	3.283	0.009^∗∗^	0.611	
HC on left DLPFC	Change of STAI-SA	Theta n.s.	–	–	–	–	–	–
HC on left DLPFC	Change of STAI-SA	Alpha n.s.	–	–	–	–	–	–
HC on DMPFC	Change of STAI-SA	Theta n.s.	–	–	–	–	–	–
HC on DMPFC	Change of STAI-SA	Alpha rACC	3.416	2.349	1.454	0.165	0.282	0.481
		Alpha precuneus	−2.923	1.019	−2.869	0.011^*^	−0.556	

rACC, rostral anterior cingulate cortex; DLPFC, dorsolateral prefrontal cortex; ITL, inferior temporal lobe; IPL, inferior parietal lobe; MDD, major depressive disorder, HC; healthy controls. ^*^p < 0.05, ^∗∗^*p* < 0.01.

**FIGURE 5:**
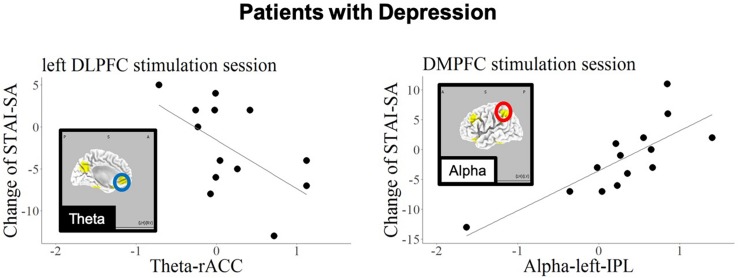
Schematic summary of results in patients with MDD, based on Table 7. The red circles enclose regions with a positive slope (*B* > 0), and best responders with negative STAI-change corresponded to decreased cortical activity. The blue circle encloses a region with a negative slope (*B* < 0), and best responders with negative STAI-change corresponded to increased cortical activity.

**FIGURE 6:**
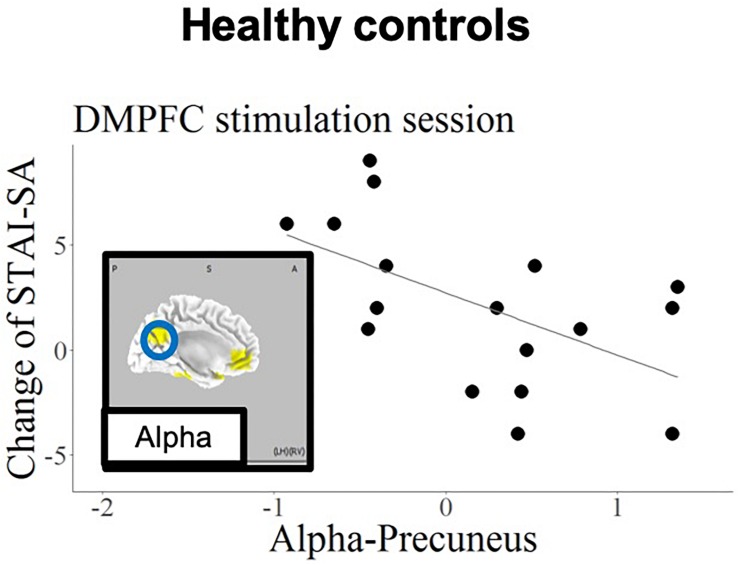
Schematic summary of results in the HC group, based on Table 7. The blue circle encloses a region with a negative slope (*B* < 0), and best responders with negative STAI-change corresponded to increased cortical activity.

Figures 5, 6 show the schematic summaries of the MDD and HC group with significantly difference (*P* < 0.05). We modeled the change in STAI-SA scores in vertical axis, and the set of cortical theta and alpha activity in 10 ROIs in horizonal axis for left DLPFC and DMPFC stimulation, and in each group.

## Discussion

The current findings confirmed that, regarding the left DLPFC stimulation site in patients with MDD, the anxiety reduction effect of tDCS was related to higher baseline theta-band activity in the rostral anterior cingulate cortex (rACC). In contrast, the anxiety reduction was associated with higher baseline alpha activity in the precuneus in the HC group.

In the current study, we have specifically focused on immediate anxiolytic effect of tDCS, and we did not expect to change sustained depressive symptoms. We indeed consider accumulation of immediate anxiolytic effects will eventually lead to long-term improvement of depressive symptoms.

The association of the anxiolytic effect of left DLPFC tDCS with high baseline theta-band activity in the rACC is in accord with the findings of previous studies (Arns et al., 2014; Li et al., 2014; Bailey et al., 2018). A large scale meta-analysis studies have further shown that functional and structural alterations in the rACC are associated with broad spectrum of psychiatric disorders (Goodkind et al., 2015; Sha et al., 2019). Patients with MDD have been reported to exhibit dysfunction in the left DLPFC as well as the rACC (Pizzagalli et al., 2002; Mayberg et al., 2005). A study for treatment resistant depression patients who were administered with rTMS for 4 to 7 weeks showed the antidepressive effect was predicted by functional connectivity between stimulation site and the subgenual cingulate (Weigand et al., 2018). Liston and colleagues also reported that activation of the subcallosal cingulate cortex was a main predictor for the effect of transcranial magnetic stimulation (Liston et al., 2014). Pizzagalli and colleagues reported that LORETA current density of theta-band in the rACC was a predictor of response to antidepressants. The current findings are in accord with these previous studies, and further suggest that the rACC, including the subcallosal cingulate cortex, is involved in the anxiolytic effects of tDCS applied to the left DLPFC, and may appear to be important for predicting the response of MDD patients to tDCS.

The current findings also revealed a correlation between baseline alpha-band activity in the IPL and state anxiety reduction during DMPFC stimulation in the MDD patient group. Several previous studies have examined the relationship between anxiety and functional brain imaging in IPL (Hasler et al., 2007; Goldin et al., 2009). Importantly, the current results revealed opposite prediction patterns in patients with MDD and HCs; the best responding HCs (exhibiting negative STAI change) were those with high alpha activity in precuneus in response to DMPFC stimulation.

A study by Fox et al. suggested that the region for stimulation by neuromodulation can be selected not only by the direct effect of the stimulation, but also by the propagation effect, depending on the interconnected regions of the resting state networks (Fox et al., 2014).

As the precuneus and ACC constitute the default mode network (Sheline et al., 2009), applying tDCS to the DMPFC might affect activity in the precuneus, which is functionally densely connected with the DMPFC. This association was only found in the HC group, presumably reflecting intact functionality of the default mode network in healthy individuals. Importantly, other remote effects of tDCS have been reported in previous studies. tDCS applied to the left DLPFC was reported to increase functional connectivity in the fronto-parietal network, while decreasing connectivity in the default mode network (van der Werf et al., 2010; Eldaief et al., 2011). It should be noted that anxiety disorder and depression are likely to be related to dysfunction of this frontal-parietal network (Sylvester et al., 2012). And this dysfunction also may yield the opposite changing of STAI-SA; decreasing the mean score of STAI-SA in MDD and increasing STAI-SA mean score in HCs.

Regarding the prediction of responses to tDCS treatment in patients with MDD, Al-Kay et al. conducted a prediction analysis with EEG data for treatment outcomes in response to prefrontal tDCS. The results revealed that frontal EEG channels were important for predicting mood improvement after treatment sessions (Al-Kaysi et al., 2017). In contrast to previous studies, the current study involved a single tDCS application, and did not examine predictors of overall treatment, but immediate responses to a single session of treatment. We believe determining the immediate neurophysiological effects of tDCS is particularly important for understanding the treatment mechanisms of tDCS, because the accumulation of immediate changes may eventually lead to long-term plasticity underlying the overall treatment effects. It should also be emphasized that sLORETA can localize activity in deeper subcortical regions, whereas scalp EEG electrodes provide limited information about the underlying cortical activity due to cortical surface orientation and volume conduction effects.

Interestingly, the patients taking no benzodiazepine medication had apparently an anxiety reduction effect for left DLPFC stimulation; however, there was no significantly difference pre-STAI scores between patients with and without benzodiazepine. The results is indeed consistent with a previous clinical trial. The clinical trial comparing the treatment response between tDCS and sertraline showed that benzodiazepine decreases the effect only in the tDCS treatment group (Brunoni et al., 2013). Benzodiazepine is used as hypnotics in this study, thus, it might be interesting to investigate interactions between tDCS and benzodiazepine relating to insomnia in future research.

Additionally, previous studies have reported slow EEG power changes before and after tDCS under the tasks, which would support further studies using slow frequencies (Keeser et al., 2011; Wirth et al., 2011; Jacobson et al., 2012; Ulam et al., 2015).

## Limitations

Our current results, demonstrating the anxiety-reducing effects of tDCS in patients with MDD, will be of interest to researchers and clinicians who seek to use neuromodulation techniques as a novel treatment for depression. However, several limitations of the current study should be noted. First, the relatively small number of participants and no placebo stimulations may warrant some caution in the interpretation of these results. Second, because we applied only one stimulation, we did not examine the therapeutic effects of tDCS on depressive symptoms. Third, chi-squared test for gender imbalance between the MDD and HC groups tended to be imbalanced, suggesting gender effect may account for the difference between MDD and HC. However, this study design also has several strengths. First, we adopted with-in subject cross over design. Second, by adopting single-session tDCS, we were able to reduce the total experiment time and burden on the subjects compared with experiments involving multiple stimulation sessions. In future research, a randomized controlled trial of tDCS intervention with a large number of participants would be helpful for addressing these limitations.

## Conclusion

The current results revealed that the immediate anxiolytic effect of left DLPFC tDCS was associated with activity in the rACC and the left IPL, whereas DMPFC stimulation was correlated with activity in the precuneus. These findings suggest that the effects of tDCS are not only directly related to the stimulation area, but also to other brain areas involved in the same resting state networks. Further, we propose that pre-stimulus EEG, in combination with the LORETA source estimation analysis, provides a promising tool for predicting the outcome of treatment intervention, including tDCS.

## Data Availability

The datasets generated for this study can be found in Kansai Medical University, KAKEN 26860950 and KAKEN 19K08056.

## Ethics Statement

The study protocol was approved by the Institutional Ethics Review Committee of Kansai Medical University.

## Author Contributions

KN and YM designed the study. KN, KK, SI, and MY recruited the participants. YK, SU, and RP-M analyzed the data. KN wrote the first draft. YM, RP-M, RI, and TK wrote the final manuscript.

## Conflict of Interest Statement

The authors declare that the research was conducted in the absence of any commercial or financial relationships that could be construed as a potential conflict of interest.
